# Evaluation of factors associated with immunoglobulin, protein, fat and lactose concentrations in colostrum of dairy cows from Austria

**DOI:** 10.1186/s13028-024-00788-0

**Published:** 2024-12-25

**Authors:** Katharina Lichtmannsperger, Nicole Hechenberger, Christina Hartsleben, Ariane Psenner, Maren Marseiler, Alexander Tichy, Thiemo Albert, Thomas Wittek

**Affiliations:** 1 Clinical Department for Farm Animals and Food System Science, Clinical Center for Ruminant and Camelid Medicine, University of Veterinary Medicine Vienna, Veterinaerplatz 1, Vienna, 1210 Austria; 2 Animal Health Service (Tiergesundheitsdienst) Salzburg, Bundesstraße 6, Wals-Siezenheim, Salzburg, 5071 Austria; 3 Department of Biological Sciences and Pathobiology, Platform for Bioinformatics and Biostatistics, Centre of Biological Sciences, University of Veterinary Medicine Vienna, Veterinaerplatz 1, Vienna, 1210 Austria; 4 Faculty of Veterinary Medicine, Institute for Food Hygiene, University of Leipzig, An den Tierkliniken 1, 04103 Leipzig, Germany

**Keywords:** Colostrum constituents, Colostrum management, Colostrum quality, Part-time farm, Simmental cows, Small scale farm, Survey

## Abstract

**Background:**

Calves rely on the passive transfer with immunoglobulins derived from colostrum. Currently, there is a lack of knowledge on colostrum management practices and colostrum quality on small scale family-owned dairy farms in Austria. The objectives of this study were to describe factors that are associated with immunoglobulin, protein, fat and lactose concentrations in dairy cow colostrum from the federal state of Salzburg. Therefore, an online questionnaire was designed to gather information on general farm characteristics. Further, the farmers collected individual colostrum samples and completed a detailed accompanying questionnaire for each sample. Immunoglobulin levels were determined by using a Brix refractometer and protein, fat and lactose by standardized laboratory methods. Linear mixed effect models were built to test factors associated with colostrum immunoglobulin, fat, protein and lactose concentrations.

**Results:**

In total, 1,050 colostrum samples from 72 dairy farms were collected. The number of calvings per year was distributed as follows: ≤10 calvings: 8.3% of the farms, 11 to 20: 31.9%, 21 to 30: 29.2%, 31 to 40: 15.3% and ≥ 41 calvings: 15.3%. Overall, the median Brix value was 22.0% (7.3–36.1%). The number of samples with good and poor-quality colostrum was 517 and 528, respectively. Cow-level factors significantly affecting colostrum Brix% were parity, calving season, *ante partum* colostrum leakage, time lag between parturition and colostrum collection. In total, a subset of 307 colostrum samples from 39 farms from pure-breed dual-purpose Simmental cows were further analysed for protein, fat and lactose concentration. The median concentration for fat was 5.1% (0.5–18.5%), protein 14.6% (4.2–27.5%) and lactose 2.3% (0.2–5.0%). The cow-level factors affecting protein concentration were similar to the factors influencing Brix%. Fat concentration was influenced by the time lag between calving and colostrum collection and by parity.

**Conclusions:**

The present study confirmed the factors, which are currently known to have an impact on colostrum quality. This was the first large scale approach in the federal state of Salzburg to survey colostrum management including colostrum sample collection. The range of colostrum quality was wide (7.3% Brix to 36.1% Brix) therefore many calves will be at risk of receiving poor quality colostrum as defined by a Brix of ≤ 22%.

**Supplementary Information:**

The online version contains supplementary material available at 10.1186/s13028-024-00788-0.

## Background

Calves rely on the passive transfer with immunoglobulins (IgGs) derived from colostrum since the cotyledonary synepitheliochorial placenta type of the cow does not allow the transfer via placenta during pregnancy. An insufficient supply with IgGs in calves is known as Failure of Transfer of Passive Immunity (FTPI) and is defined by a low concentration of IgGs in the blood. At the herd-level, > 40% of the calves should show excellent serum IgG levels of ≥ 25 g/L and only < 10% of the calves should show poor serum IgG levels of < 10 g/L [1]. FTPI leads to higher morbidity and mortality rates and therefore poses a major animal welfare issue [2]. In a meta-analysis the risk to suffer from neonatal disease, such as diarrhoea (OR = 1.51) and bovine respiratory disease (BRD) (OR = 1.75), was increased in calves showing FTPI [2]. Additionally, a study carried out by our research group concluded that calves showing diarrhoea in the first three weeks of life were associated with having FTPI (OR = 2.69) [3]. Feeding a high quantity (> 4 L) of good quality (Brix > 22%) colostrum immediately after parturition is the most important recommendation to prevent FTPI. For an excellent colostrum supply, calves need to receive greater than or equal to 300 g of IgG shortly after birth [1]. There are multiple management-, cow- and environment-related factors that have been described to have a significant effect on colostrum quality in terms of IgG concentration. The time lag between parturition and first milking is one of the most critical points to harvest good quality colostrum as it has been described that colostrum IgG concentration decreases by 3.7% per hour after parturition [4]. The timing of colostrum harvest is an important colostrum management practice which can easily be influenced by the farmer [5]. Additionally, colostrum bacterial contamination can be controlled with implementing good colostrum management practices via arranging an optimal harvesting and storing hygiene. The goals for good quality colostrum in terms of bacterial contamination are total plate counts (TPC) of less than 100,000 colony forming units (cfu)/mL and coliform counts (TCC) of less than 10,000 cfu/mL, respectively [6]. Calves receiving colostrum severely contaminated with bacteria showed a decreased apparent efficiency of absorption versus calves receiving colostrum with low bacteria counts. Bacteria reduction can be achieved by heat-treatment/pasteurization of colostrum [7]. The major source of colostrum contamination is an unhygienic harvesting process and/or inadequate storage conditions [8]. An investigation carried out on grassland-based dairy systems found that almost 81% of the colostrum samples did not meet the industry guidelines for bacterial contamination [9]. Another management-related factor that has an impact on IgG concentration is the dry-period length although findings are inconsistent [10, 11]. The temperature humidity index (THI) in the far-off (dry-off until three weeks before calving) and close up (approx. three weeks before calving) dry-period has been proven to have a significant effect on colostrum quality but also with inconsistent findings [10, 12]. Recent studies showed that the presence of the calf during colostrum harvest resulted in a higher IgG concentration in colostrum [13]. Cow-related factors, such as the number of lactations, previous 305-day milk yield, genetic parameters, *ante partum* milk leakage, colostrum quantity, the metabolic status of the cow and udder health in terms of somatic cell counts has been described to influence colostrum quality [5, 10, 14–17]. There is a strong herd-level variation between colostrum quality, primarily depending on the implemented herd-level management procedures (e.g. volume fed at first meal, colostrum quality, time-lag between parturition and first feeding) which subsequently affects the number of calves showing FTPI [18, 19]. Beside IgGs, colostrum contains multiple other essential constituents such as cytokines, growth factors, mRNA, oligosaccharides, maternal leucocytes, vitamins, minerals, hormones, non-specific antimicrobial factors and nutrients [20]. In contrast to whole milk, colostrum from cows shows a higher total solid percentage (colostrum = 23.9% versus whole milk = 12.9%) and contains higher protein and fat concentrations 14.0% and 6.7%, respectively [21]. The protein fraction consists of albumin, casein and IgGs. The Ig fraction can be further categorized in IgG1 and IgG2 (~ 85–90%), IgA (≤ 5%) and IgM (≤ 7%) whereof IgGs are the most abundant ones [1]. Fat but also the fat-soluble vitamins are considerably concentrated in bovine colostrum in comparison to whole milk and transition milk [1]. The concentration range of fat in colostrum has been reported to be wide with 6.7 ± 4.2% (2.0–26.5%) from a study in Pennsylvania including 55 Holstein dairy farms and 5.6 ± 3.2% (1.0–21.7%) from a study carried out in 12 US states including 67 farms with different breeds [22, 23]. Fat in colostrum is an important energy source for the newborn since the body fat reserves are limited [23]. Recent literature showed that also the fatty acid profile differs between cow parity and whole milk, transition milk and colostrum [24]. Lactose concentration is low in colostrum and changes in an inverse manner to other constituents such as fat, protein and ash [22]. Lactose is responsible for 50% of the osmotic pressure of milk and therefore is responsible for the influx of water into milk which regulates the volume produced [25]. The aims of this study were to describe factors that are associated with IgG, protein, fat and lactose concentration in dairy cow colostrum from the Austrian federal state of Salzburg. We hypothesized that herd-level and cow-level factors are significantly associated with colostrum IgG, protein, fat and lactose concentrations from dairy cows.

## Methods

### Ethical consideration

This study was approved by the Ethics and Animal Welfare Committee of the University of Veterinary Medicine, Vienna. Since the study did not include invasive measures, a governmental approval was not required.

### Online survey

All members (*n* = 1,747 dairy farms/cow-calf operations) of the Animal Health Service Salzburg, Austria (Tiergesundheitsdienst Salzburg) received an invitation via e-mail to participate in the study. An online questionnaire was designed using Google Forms. The questionnaire included questions on general farm characteristics including the number of calvings per year (farm size), part-time or full-time farmer (operation type), organic or conventional production (production regime). Additionally, information on colostrum management was gathered. The questionnaire on general farm characteristics (federal state, member of the animal health service, member of the national breeding association, dairy or cow-calf operation, operation type, production type, number of cows, livestock units, breed, housing system, 305-day milk yield) and general on-farm colostrum management practices (first milking, cow calf separation, udder cleaning method, colostrum harvest technique, calf feeding, amount of colostrum fed and feeding practices, colostrum from the dam, quality assessment,) were primarily single choice questions. The questionnaire has been published by our group [26] and was only slightly modified. All farmers participating in the online questionnaire had the opportunity to participate actively in the study which meant to collect colostrum samples from dairy cows on their respective farms.

### Farms and animals

In total, 72 farms from five districts of the federal state Salzburg, Austria, participated actively in the study. The farms were in the districts of Tennengau (*n* = 4), Lungau (*n* = 7), Pinzgau (*n* = 14), Flachgau (*n* = 28) and Pongau (*n* = 19). Forty-six (63.9%) organic producing farms and 26 (36.1%) conventional farms were included. Full-time farmers (*n* = 41; 55.6%) and part-time farmers (*n* = 31; 44.4%) were included, respectively. The number of calvings per year was distributed as follows: ≤10 calvings: 6 farms, 11 to 20 calvings: 23 farms, 21 to 30 calvings: 21 farms, 31 to 40 calvings: 11 farms and ≥ 41 calvings: 11 farms. Thirty-five (48.6%) farms kept pure-breed Simmental cows, 3 (4.2%) farms kept pure-breed Pinzgauer cows, and 2 (2.8%) farms kept pure-breed Holstein cows. The remaining 32 farms had a mixed herd with additionally Brown Swiss, Holstein or other breeds such as Montbéliard, Jersey and Normande. Details on colostrum sample collection, storage and logistics are provided in the Additional File 1.

### Brix%

The IgG concentrations were measured at room temperature by one of the authors indirectly using a digital Brix refractometer (0 to 85% Brix; HM-DREF-1^®^, Hebesberger Messtechnik, Neuhofen, Austria) in the diagnostic laboratory of the Clinical Center for Ruminant and Camelid Medicine. The detailed procedure has been described elsewhere [3]. In brief, the refractometer was calibrated using deionized water and subsequently the colostrum sample was measured. Good quality colostrum was defined as a Brix value of > 22% and poor quality colostrum of a Brix value ≤ 22% [27].

### Bacterial contamination

Bacterial contamination was expressed TPC and TCC. In the diagnostic laboratory at first a 1:10 dilution series was prepared and subsequently the dilutions (10^ − 1^, 10 ^− 2^, 10 ^− 3^) were plated on Columbia agar (with 5% sheep blood) and MacConkey agar for the assessment of TPC and TCC, respectively. If the bacterial contamination was too high, exceeding 300 cfu/mL, an additional dilution was prepared (dilution = 10^− 4^). The detailed procedure has been described elsewhere [3]. The samples were categorized as low bacterial counts colostrum using the thresholds of 100,000 cfu/mL and 10,000 cfu/mL for TPC and TCC, respectively [6]. All colostrum samples which showed high cfu (> 300 cfu/plate) in at least two plates they were assessed as “elevated” (exceeding the thresholds of TPC > 100,000 cfu/mL and TCC > 10,000 cfu/mL) and not included for calculating the minimum, maximum and percentiles of TPC and TCC, respectively. At least two plates of the dilution series need to meet the inclusion criteria of < 300 cfu/mL respectively to have a countable number of colonies (e.g. dilution not 1:10 = unreliable result) otherwise they were categorized as “not assessable = n. a.”.

### Protein, fat and lactose concentrations

#### Selection of colostrum samples

In total, 300 of the 1,050 investigated colostrum samples were randomly selected for further analysis of colostrum protein, fat and lactose concentration. Seven additional samples were included (*n* = 307) in case of unexpected errors due to the colostrum viscosity. The first randomization step was to quantify the absolute and the relative number of the dairy cows per district (Salzburg/Salzburg Stadt, Tennengau, Pinzgau, Pongau, Lungau) in the federal state of Salzburg. Subsequently, the number of selected colostrum samples per district were chosen based on this outcome. Another inclusion criteria was the membership of the National Milk Recording Association (Landeskontrollverband LKV) and that the cows were pure-breed dual-purpose Simmental breed. In total 681 dairy cows from 43 dairy farms met the inclusion criteria and were used as a pool for random sample size calculations. All samples were divided into the districts and the production regime (organic versus conventional). A random number between 0 and 1 was generated by using the “=rand()” function of Microsoft Excel for each colostrum sample. These numbers were sorted, and the first samples were chosen. In the district of Tennengau and Lungau, all samples originated from organic farms (Tennengau: 19 samples from 2 farms, Lungau: 72 sample from 3 farms). In Pinzgau (11 farms), 44 samples originated from organic farms and 37 from conventional farms. In Flachgau/Salzburg Stadt (13 farms), 64 samples originated from organic and 64 samples from conventional farms, and in Pongau (14 farms), 44 samples originated from organic and 37 samples from conventional farms.

#### Laboratory procedure

In total, 307 bovine colostrum samples from 39 dairy farms were analyzed for fat, protein and lactose concentrations according to the methods described elsewhere [28]. For fat, protein and lactose an internal quality control was implemented and 26, 42 and 7 colostrum samples were tested as duplicates, respectively. The internal quality control showed a median deviation between the two measured values of 0.21% (min=-13.72%, max = 2.8%, 25th percentile=-0.33%, 75th percentile = 0.86%), 0.0% (min=-0.01%, max = 0.17%, 25th percentile = 0.0%, 75th percentile = 0.0%) and − 0.52% (min=-1.58%, max = 2.21%, 25th percentile=-0.52%, 75th percentile=-0.52%) for fat, protein and lactose values, respectively.

### Statistical analysis

Data was collected and summarized in Microsoft Excel 2016. The complete data set was transferred to IBM^®^ SPSS^®^ Statistics Version 29 (IBM^®^, New York, USA) for further statistical analysis. The online questionnaire (*n* = 72 farms) was coded and the laboratory results (*n* = 1,050 samples including accompanying questionnaire) were included. The laboratory results for Brix% and plate counts, fat, protein and lactose concentrations were used to calculate the median, range (minimum, maximum) and the percentiles. The definition of an extreme outlier was: >75th percentile adding three times the interquartile range. The Kolmogorov-Smirnov test including the Lilliefors correction was implemented to test for normality. Since the Brix values were not normally distributed the Spearman Rank correlation was implemented to test the correlation between colostrum Brix% and colostrum protein concentration. The significance level was set at *P* < 0.05. Linear mixed effect models were built to test factors associated with colostrum IgG, fat, protein and lactose concentrations. Therefore, the continuous variables Brix% (estimates IgG), fat%, protein% and lactose% were used as dependent variables. In the model, farm ID was implemented as subject and the sample ID as repeated measure. Associations were calculated at the herd-level and farm size, operation type, production regime, district and udder cleaning before colostrum harvest were used as fixed effects. As a post hoc test, the Sidak test was implemented. On the cow-level following factors were included as fixed effects: the season of calving, time of calving (day, night), the lactation number (1st, 2nd, 3rd, 4th, 5th, 6th, >6th), the dry period length (< 8 weeks, 8–12 weeks, > 12 weeks), dry off procedure (antibiotic treatment, internal teat sealant = ITS, no medication), disease during the dry period (yes/no), colostrum leakage (yes/no), vaccination (yes/no), time to colostrum harvest (≤ 120 min, 121 to 360 min, > 360 min) quantity of harvested colostrum (0 to 3 L, 4 to 6 L, > 6 L), udder cleaning before colostrum harvest (yes/no), Total plate counts (< 100,000 cfu/mL, not assessable, ≥ 100,000 cfu/mL) and coliform counts (< 10,000 cfu/mL, not assessable ≥ 10,000 cfu/mL). The best model was selected according to the Akaike’s Information Criterion (AIC). The factor “season of calving” was summarized as follows: (winter = December, January, February; spring = March, April, May; summer = June, July, August; autumn = September, October, November). All lactations > 6 (7th to the 14th lactation) were summarized to one variable. The factor “time” was categorized in parturition during night (10:00 p.m. to 6:00 a.m.) or during the day (06:01 a.m. to 09:59 p.m.).

## Results

### Colostrum management practices

This section shows the results from the online survey prior to colostrum sample collection. In total, 54.2% of the 72 included farms harvested colostrum within one hour after calving, 38.9% within six hours, 2.8% stated that the calf stayed with the dam meaning the calf harvested colostrum through suckling and 4.2% milked the cow when the next milking took place (day or evening milking). The majority, 59.7% answered that they use a milking machine to collect colostrum, 36.1% harvested colostrum by hand and on 4.2% farms, the calf stayed with the dam. Regarding colostrum quality check before feeding, 9.7% of the 72 farmers used a device to test colostrum quality using a colostrometer (*n* = 1), a refractometer (*n* = 3), a funnel (*n* = 2) or a funnel and refractometer (*n* = 1). Fifty-eight farms had a frozen colostrum stock and 19.4% do not. Sixty-one always fed the colostrum of the mother to her own calf, 6.9% only fed the colostrum from the mother if it was of good quality, on4.2% of the farms the calf stayed with the mother and 4.2% answers were invalid due to contradiction. Thirty-nine farms fed colostrum to the calf within one hour after calving, 37.5% of the farms within six hours, 4.2% left the calf suckling colostrum from the dam and 4.2% fed the calf after the next routine milking (morning or evening milking). The majority, 70.8% of the farms used a feeding bucket to feed colostrum, 25.0% used a nipple bottle, 2.8% left the calf with the dam and 1.4% used an esophageal tube. If the calf was not drinking, 66.7% fed the calf a second time two to four hours later, 9.7% immediately drenched the calf using an esophageal tube, 22.2% fed the calf at the next milking and 1.4% answered that they would stay with the calf until it starts drinking.

### Colostrum samples

In total, 1,050 individual colostrum samples from primiparous (*n* = 278) and multiparous (*n* = 751) cows were collected, in 21 cases there was no information on lactation number. The mean number was 15 samples per farm (min = 1, max = 54). The median time lag between parturition (*n* = 1,017 answers) and colostrum harvest was 60 min (min = 0 min, max = 1,260 min, 25th percentile = 30 min, 75th percentile = 180 min). The median time lag between parturition and calf feeding (*n* = 997 answers) was 75 min (min = 0 min, max = 1,320 min, 25th percentile = 35.0 min, 75th percentile = 182.5 min).

### Brix%

**Fig. 1:**
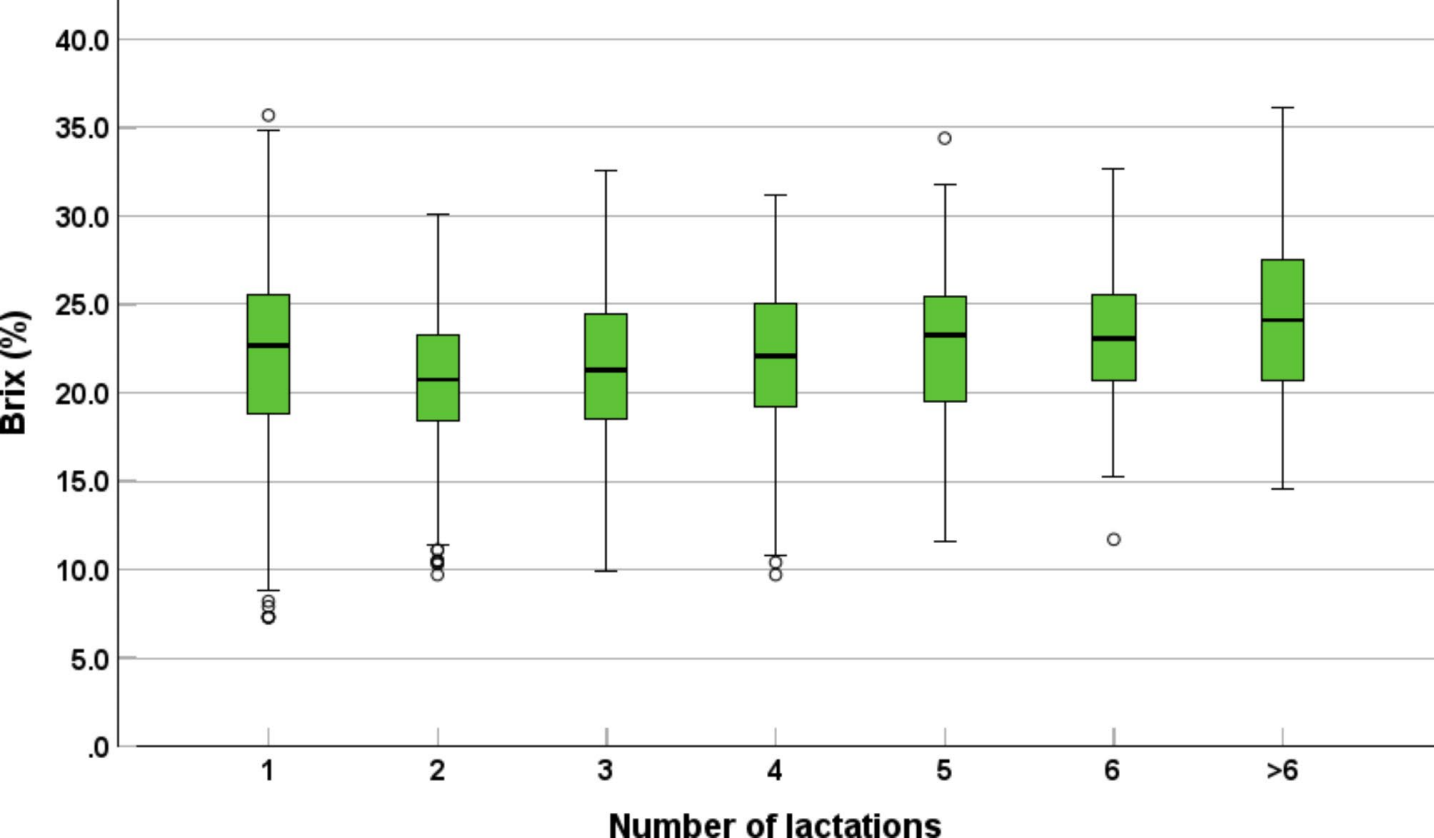
Overview of colostrum quality in terms of Brix%. In total, 1,045 samples originating from 72 farms in the federal state of Salzburg, Austria were included. On average 15 samples were collected per farm (min = 1, max = 54). The Brix% of the cows in the first (*n* = 276), second (*n* = 224), third (*n* = 176), fourth (*n* = 115), fifth (*n* = 88), sixth (*n* = 62) and > 6 lactations (*n* = 83) are shown

In total, 1,045 of the 1,050 examined samples provided a readable result, five samples were excluded due to technical problems. The median Brix value was 22.0% (min = 7.3%, max = 36.1%, 25th percentile = 19.0%, 75th percentile = 25.1%). The number of samples with good and poor-quality colostrum was 517 (49.5%) and 528 (50.5%), respectively. The cows in the first (*n* = 276), second (*n* = 224), third (*n* = 176), fourth (*n* = 115), fifth (*n* = 88), sixth (*n* = 62) and > 6 (*n* = 83) lactations showed median Brix% values of 22.7%, 20.8%, 21.3%, 22.1%, 23.3%, 23.1% and 24.1%. For details see Fig. 1.

### Bacterial contamination

Of the 668 samples included for TPC analysis, 325 (48.7%) revealed < 100,000 cfu/mL, 141 (21.1%) were not assessable and 202 (30.2%) were ≥ 100,000 cfu/mL. Of the 670 samples investigated for TCC, 577 (86.1%) showed < 10,000 cfu/mL, 53 (7.9%) were not assessable and 40 samples (3.8%) showed ≥ 10,000 cfu/mL. In total, 380 and 592 colostrum samples were counted TPC and TCC per millilitre, respectively. The median TPC and TCC were 17,825 cfu/mL (min = 0, max = 461,000.0, 25th percentile = 4,225.0, 75th percentile = 44,037.5) and 0.0 cfu/mL, respectively.

### Protein, fat and lactose concentration

**Fig. 2:**
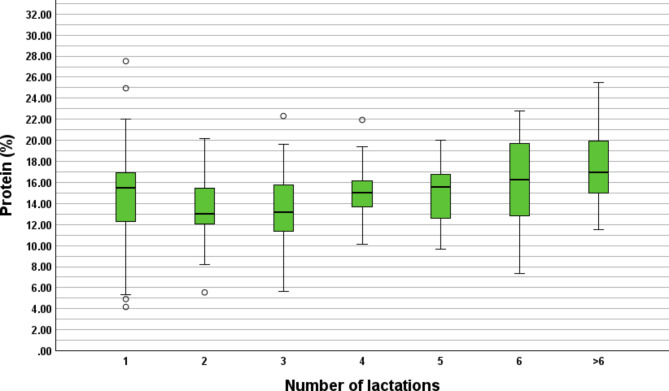
Overview of colostrum protein concentration of the 307 further investigated samples from Simmental dairy cows. The samples were from 39 dairy farms from five different districts of the federal state of Salzburg, Austria. The protein% of the cows in the first (*n* = 89), second (*n* = 68), third (*n* = 51), fourth (*n* = 32), fifth (*n* = 27), sixth (*n* = 14) and > 6 lactation (*n* = 26) are shown. Extreme outliers are shown as asterisk

**Fig. 3:**
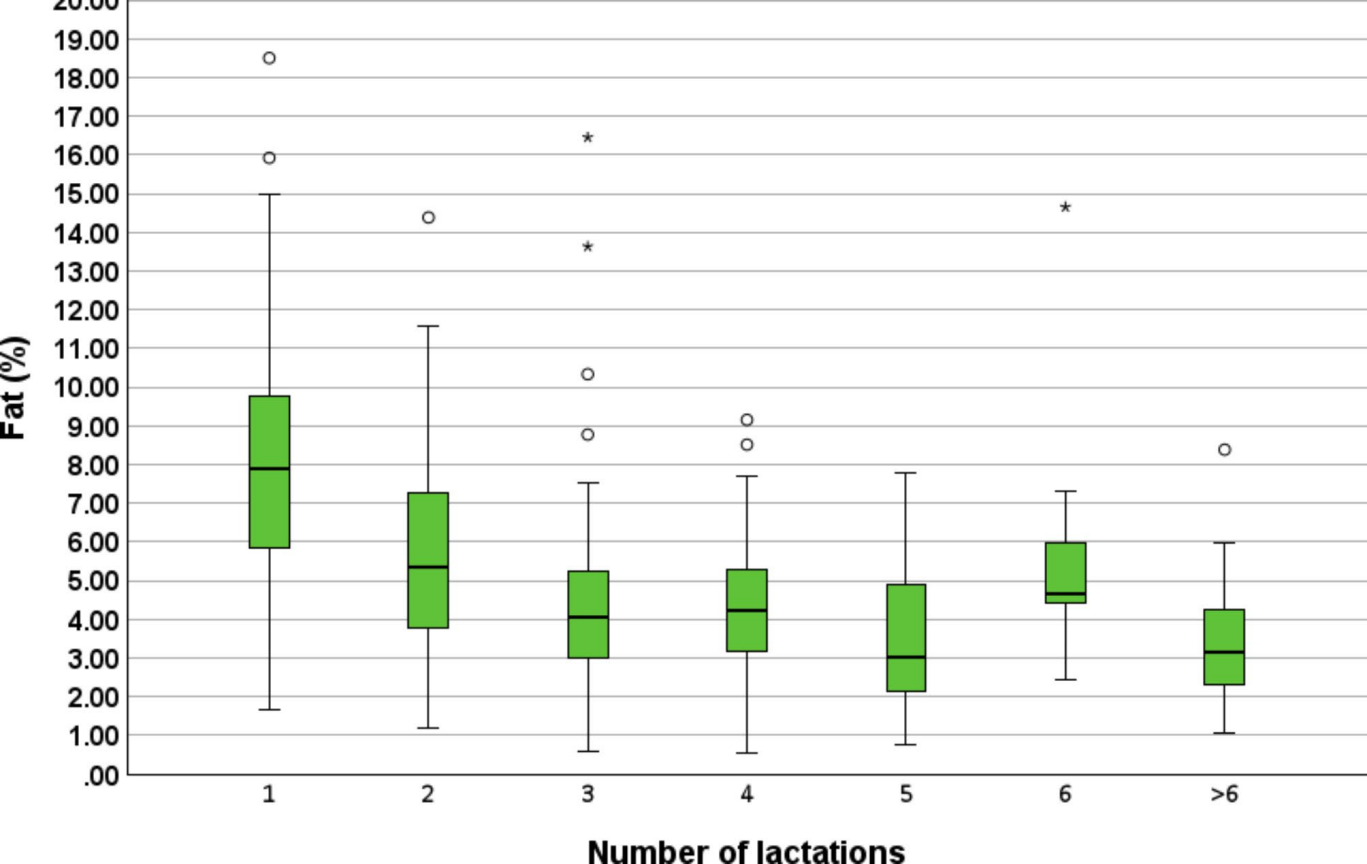
Overview of colostrum fat concentration of the 307 further investigated samples from Simmental dairy cows. The samples were from 39 dairy farms from five different districts of the federal state of Salzburg, Austria. The fat% of the cows in the first (*n* = 89), second (*n* = 68), third (*n* = 51), fourth (*n* = 32), fifth (*n* = 27), sixth (*n* = 14) and > 6 lactations (*n* = 26) are shown. Extreme outliers are shown as asterisk

**Fig. 4:**
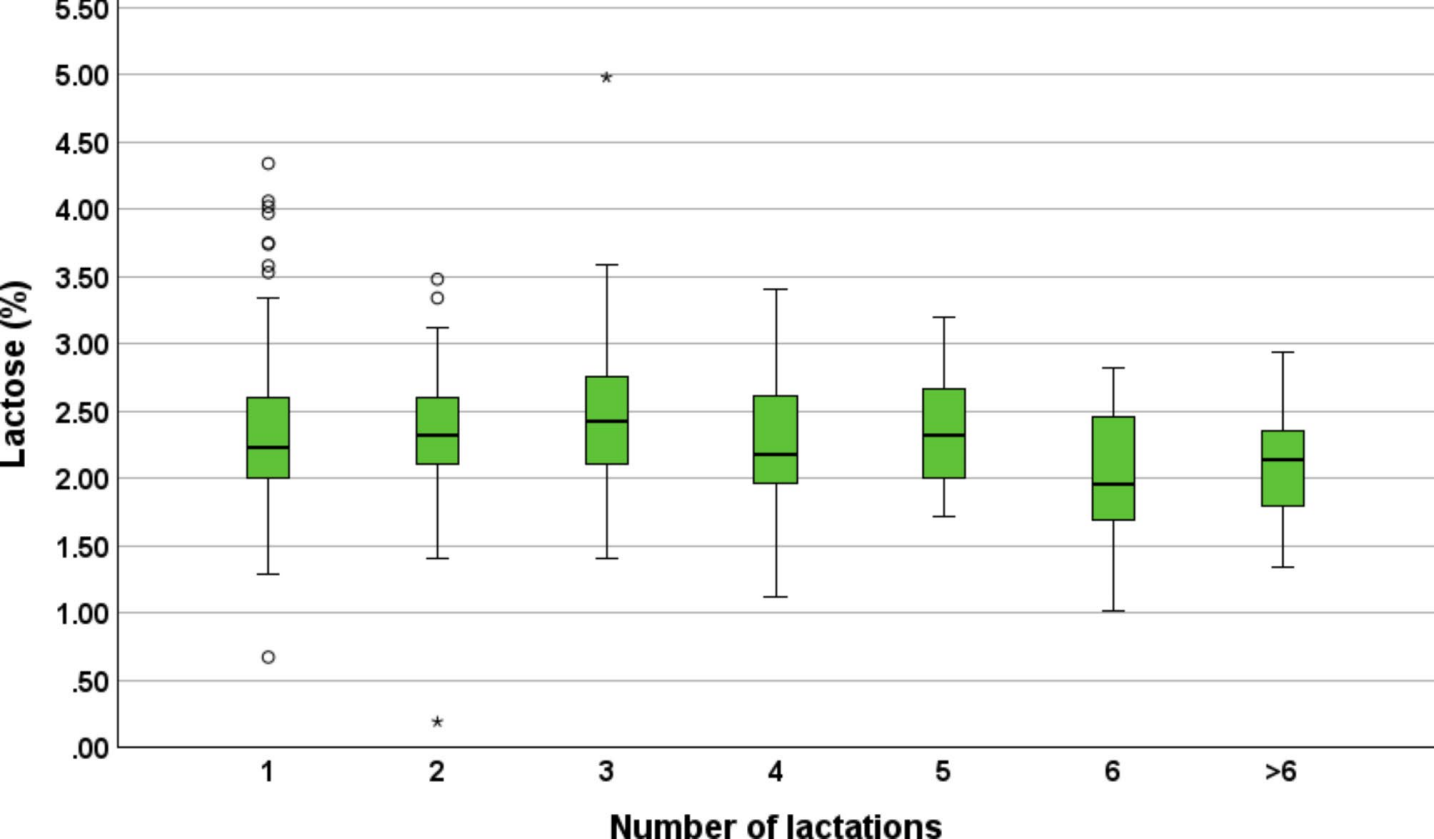
Overview of colostrum lactose concentration of the 307 further investigated samples from Simmental dairy cows. The samples were from 39 dairy farms from five different districts of the federal state of Salzburg, Austria. The lactose% of the cows in the first (*n* = 89), second (*n* = 68), third (*n* = 51), fourth (*n* = 32), fifth (*n* = 27), sixth (*n* = 14) and > 6 lactation (*n* = 26) are shown. Extreme outliers are shown as asterisk

In total, 307 colostrum samples from 39 farms were examined for colostrum protein, fat and lactose concentration. The cows were in their 1st (*n* = 89), 2nd (*n* = 68), 3rd (*n* = 51), 4th (*n* = 32), 5th (*n* = 27), 6th (*n* = 14) or > 6 lactation (*n* = 26). The correlation between the Brix% and protein concentration was *r* = 0.91 (*P* < 0.01), Brix% and fat concentration *r* = 0.29 (*P* < 0.01) and Brix% and lactose concentration *r*=-0.66 (*P* < 0.01). Overall, the 307 examined colostrum samples from Simmental dairy cows showed a median fat concentration of 5.1% (min = 0.5%, max = 18.5%, 25th percentile = 3.3%, 75th percentile = 7.4%), protein concentration of 14.6% (min = 4.2%, max = 27.5%, 25th percentile = 12.3%, 75th percentile = 16.7%) and lactose concentration of 2.3% (min = 0.2%, max = 5.0%, 25th percentile = 2.0, 75th percentile = 2.6%). Cows in their 1st to > 6 lactations showed median fat, protein and lactose percentages from 3.0 to 7.9%, 13.1–17.0% and 2.0–2.4%, respectively. Protein, fat and lactose concentrations in regard to the number of lactations are shown in the Boxplots Figs. 2, 3 and 4. Additionally, further information is provided in Additional File 2.

### Factors affecting Brix%, protein, fat and lactose concentrations

#### Herd-level factors affecting Brix%

At the herd-level, there were differences between farms with ≥ 41 calvings per year in comparison to farms with 31 to 40 calvings (*P* < 0.01) and 21 to 30 calvings (*P* = 0.02). Colostrum quality was significantly lower in farms with ≥ 41 calvings; on average − 1.09% Brix in comparison to farms with 21 to 30 calvings and − 2.16% Brix in farms with 31 to 40 calvings. Regarding the udder cleaning methods, the farms which did not clean the udders before colostrum harvest showed significantly lower colostrum qualities (-1.72% Brix) (*P* = 0.01). Farms located in the district of Pongau appeared with significantly lower quality in comparison to the district of Lungau (-3.06% Brix) (*P* < 0.01), Pinzgau (-1.98% Brix) (*P* < 0.01) and Flachgau/ Salzburg Stadt (-1.13% Brix) (*P* < 0.01). Regarding the production type, colostrum samples originating from organic farms showed significantly lower colostrum quality (-1.46% Brix) than colostrum harvested from cows on conventional farms (*P* < 0.01). Regarding the operation type, colostrum samples originating from full-time farms revealed significantly lower colostrum quality (-0.95% Brix) than colostrum harvested from cows on part-time farms (*P* = 0.03). The results are summarized in Tables 1 and 2.

**Table 1:** Overview on the investigated herd-level factors affecting colostrum quality. The 1,050 samples were collected from 72 dairy farmers from the federal district of Salzburg, Austria. The Brix% was evaluated in the diagnostic lab of the Clinical Centre for Ruminant and Camelid Medicine, Vienna by using a digital Brix refractometer. Additional information (herd-level factors) was gathered by an online questionnaire and an accompanying questionnaire

Herd-level Category	Factor	N total farms	N total samples	Brix ≤ 22%		Brix > 22%	
				N cows	N%	N cows	N%
Farm size	≤ 10	6	35	18	51.4%	17	48.6%
	11 to 20	23	233	113	48.5%	120	51.5%
	21 to 30	21	262	138	52.7%	124	47.3%
	31 to 40	11	220	92	41.8%	128	58.2%
	≥ 41	11	295	167	56.6%	128	43.4%
Udder cleaning before colostrum harvest	No	8	67	48	71.6%	19	28.4%
	Yes	64	978	480	49.1%	498	50.9%
Districts	Tennengau	4	39	18	46.2%	21	53.8%
	Lungau	7	108	38	35.2%	70	64.8%
	Pinzgau	14	159	76	47.8%	83	52.2%
	Flachgau/SalzburgStadt	28	461	237	51.4%	224	48.6%
	Pongau	19	278	159	57.2%	119	42.8%
Production regime	Conventional	25	400	169	42.3%	231	57.8%
	Organic	47	645	359	55.7%	286	44.3%
Operation type	Full-time farmer	41	706	370	52.5%	335	47.5%
	Part-time farmer	31	339	158	46.5%	182	53.5%

**Table 2:** Overview on the differences in colostrum quality in terms of Brix% at the herd-level. The 1,050 samples were collected from 72 dairy farmers from the federal district of Salzburg, Austria. The Brix% was evaluated in the diagnostic lab of the Clinical Centre for Ruminant and Camelid Medicine Vienna by using a digital Brix refractometer. Additional information (herd-level factors) was gathered by an online questionnaire and an accompanying questionnaire

Herd-level category	Factor	Mean Brix%	95% Confidence Interval		P value
			Lower CI	Upper CI	
Farm size	≤ 10	21.73	19.94	23.51	< 0.01*
	11 to 20	21.54	20.60	22.48	
	21 to 30 ^a^	21.90	21.05	22.74	
	31 to 40 ^b^	22.97	22.01	23.92	
	≥ 41 ^a, b^	20.80	19.97	21.64	
Udder cleaning before colostrum harvest	No	20.93	19.67	22.18	0.01*
	Yes	22.64	22.17	23.12	
Districts	Tennengau	21.67	20.05	23.30	< 0.01*
	Lungau ^a, b^	23.33	22.17	24.49	
	Pinzgau ^c^	22.25	21.30	23.19	
	Flachgau/SalzburgStadt^ b, d^	21.40	20.64	22.16	
	Pongau ^a, c,d^	20.27	19.57	20.98	
Production regime	Conventional	22.51	21.67	23.36	< 0.01*
	Organic	21.06	20.34	21.78	
Operation type	Full-time farmer	21.31	20.48	22.14	0.03*
	Part-time farmer	22.26	21.39	23.13	

Significant P values (*P* < 0.05) are highlighted with an asterisk *. Herd-level factors which were significantly different within the category are highlighted with superscript letters

#### Cow-level factors affecting Brix%

There was a statistically significant difference regarding the season of colostrum sampling. Colostrum samples obtained in autumn showed a higher quality in comparison to colostrum samples gathered during winter (mean Brix% within season=-1.43% Brix) (*P* = 0.18), summer (-2.63% Brix) (*P* < 0.01) and spring (-2.33% Brix) (*P* < 0.01. *Ante partum* milk leakage was found to be a significant cow-level effect on colostrum quality whereof the cows showing milk leakage had a lower colostrum quality (-1.45% Brix) (*P* < 0.01). Colostrum harvested > 360 min after calving showed a significantly lower quality in comparison to colostrum harvested within the first 120 min after calving (-3.82% Brix) (*P* < 0.01) and 121 to 360 min after birth (-2.92% Brix) (*P* < 0.01). No significant effect was reported for the factor “time of calving” (*P* = 0.60), “dry period length” (*P* = 0.59), “dry-off procedure” (*P* = 0.99), “disease during the dry period” (*P* = 0.48), “vaccination of the dam” (*P* = 0.68), “quantity of colostrum harvested” (*P* = 0.44), “colostrum total plate counts” (*P* = 0.18) and “coliform counts” (*P* = 0.28). The results are summarized in Tables 3 and 4.

**Table 3:** Overview of the investigated cow-level factors influencing colostrum quality. The 1,050 samples were collected from 72 dairy farmers from the federal district of Salzburg, Austria. The Brix% was evaluated in the diagnostic lab of the Clinical Centre for Ruminants and Camelid Medicine, Vienna by using a digital Brix refractometer. Additional information (herd-level factors) was gathered by an online questionnaire and an accompanying questionnaire

Cow-level Category	N total samples	Details	N samples	Brix ≤ 22%		Brix > 22%	
				N cows	N%	N cows	N%
Season of calving	1,045	Winter	366	190	51.9%	176	48.1%
		Spring	216	109	50.5%	107	49.5%
		Summer	185	116	62.7%	69	37.3%
		Autumn	278	113	40.6%	165	59.4%
Time of calving	1,041	Day	666	334	50.2%	332	49.8%
		Night	343	173	50.4%	170	49.6%
Lactation number	1,024	1st lactation	276	122	44.2%	154	55.8%
		2nd lactation	224	147	65.6%	77	34.4%
		3rd lactation	176	98	55.7%	78	44.3%
		4th lactation	115	57	49.6%	58	50.4%
		5th lactation	88	36	40.9%	52	59.1%
		6th lactation	62	25	40.3%	37	59.7%
		> 6 lactations	83	28	33.7%	55	66.3%
Dry period length	1,013	Primiparous	276	122	44.2%	154	55.8%
		< 8 weeks	250	127	50.8%	123	49.2%
		8 to 12 weeks	439	245	55.8%	194	44.2%
		> 12 weeks	48	16	33.3%	32	66.7%
Dry off procedure	994	Primiparous cow	273	123	45.1%	150	54.9%
		Antibiotic treatment	373	186	49.9%	187	50.1%
		ITS	153	74	48.4%	79	51.6%
		No medication	176	103	58.5%	73	41.5%
		Others	19	12	63.2%	7	36.8%
Disease during the dry period	1,008	Yes	32	19	59.4%	13	40.6%
		No	968	477	49.3%	491	50.7%
		Prophylaxis	8	7	87.5%	1	12.5%
Colostrum leakage	1,016	Yes	200	114	57.0%	86	43.0%
		No	816	394	48.3%	422	51.7%
Vaccination dam	1,013	Yes	141	65	46.1%	76	53.9%
		No	872	444	50.9%	428	49.1%
Time to colostrum harvest	1,010	≤ 120 min	671	315	46.9%	356	53.1%
		121 to 360 min	258	135	52.3%	123	47.7%
		> 360 min	81	59	72.8%	22	27.2%
Quantity colostrum harvest	1,013	0 to 3 L	508	224	44.1%	284	55.9%
		4 to 6 L	405	227	56.0%	178	44.0%
		> 6 L	100	58	58.0%	42	42.0%
Udder cleaning before colostrum harvest	1,017	Yes	922	469	50.9%	453	49.1%
		No	95	41	43.2%	54	56.8%
Total plate counts	667	< 100,000 cfu/mL	325	150	46.2%	175	53.8%
		not assessable	141	70	49.6%	71	50.4%
		≥ 100,000 cfu/mL	201	101	50.2%	100	49.8%
Coliform counts	669	< 10,000 cfu/mL	576	274	47.6%	302	52.4%
		not assessable	53	25	47.2%	28	52.8%
		≥ 10,000 cfu/mL	40	23	57.5%	17	42.5%

Winter: December, January, February; Spring: March, April, May; Summer: June, July, August; Autumn: September, October, November; Day: 06:01 a.m. to 21:59 p.m.; Night: 10:00 p.m. to 06:00 a.m.; >6 lactations: 7th to 14th lactation; ITS: internal teat sealant, cfu: colony forming units

**Table 4:** Overview on the differences in colostrum quality at the cow-level. The 1,050 samples were collected from 72 dairy farmers from the federal district of Salzburg, Austria. The Brix% was evaluated in the diagnostic lab of the Clinical Centre for Ruminant and Camelid Medicine, Vienna by using a digital Brix refractometer. Additional information (herd-level factors) was gathered by an online questionnaire and an accompanying questionnaire

Cow-level category	Factor	Mean Brix%	95% Confidence Interval		P value
			Lower	Upper	
Season of calving	Winter	20.70	18.95	22.46	0.02*
	Spring ^a^	19.81	18.01	21.61	
	Summer ^b^	19.50	17.45	21.56	
	Autumn ^a, b^	22.14	20.20	24.07	
Time of calving	Night	20.66	18.85	22.47	0.60
	Day	20.42	18.68	22.15	
Lactation number	2nd lactation ^a, b^	18.96	17.14	20.79	< 0.01*
	3rd lactation^c^	19.68	17.85	21.50	
	4th lactation	19.98	18.04	21.92	
	5th lactation	20.83	18.76	22.91	
	6th lactation ^a^	21.70	19.65	23.75	
	> 6 lactations ^b, c^	22.08	19.96	24.20	
Dry period length	< 8 weeks	20.73	18.95	22.52	0.59
	8 to 12 weeks	20.30	18.59	22.01	
	> 12 weeks	20.58	18.25	22.91	
Dry off procedure	Antibiotic treatment	20.49	18.85	22.13	0.99
	ITS	20.47	18.65	22.28	
	No medication	20.52	18.73	22.32	
	Others	20.68	17.85	23.51	
Disease during the dry period	Yes	20.15	17.64	22.67	0.48
	No	20.92	19.56	22.29	
Colostrum leakage	Yes	19.81	17.95	21.67	0.02*
	No	21.27	19.58	22.95	
Vaccination dam	Yes	20.67	18.61	22.73	0.68
	No	20.40	18.83	21.98	
Time to colostrum harvest	≤ 120 minutes^a^	22.11	20.41	23.81	< 0.01*
	121 to 360 minutes^b^	21.21	19.41	23.01	
	> 360 minutes^a, b^	18.29	16.15	20.43	
Quantity colostrum harvest	0 to 3 L	20.93	19.17	22.68	0.44
	4 to 6 L	20.50	18.77	22.22	
	> 6 L	20.19	18.15	22.24	
Total plate counts	< 100,000 cfu/mL	20.68	18.88	22.47	0.18
	not assessable	20.00	18.14	21.85	
	≥ 100,000 cfu/mL	20.94	19.16	22.73	
Coliform counts	< 10,000 cfu/mL	20.95	19.40	22.49	0.28
	not assessable	21.15	19.07	23.24	
	≥ 10,000 cfu/mL	19.51	17.15	21.88	

Significant P values (*P* < 0.05) are highlighted with an asterisk *. Herd-level factors which were significantly different within the category are highlighted with superscript letters. Winter: December, January, February; Spring: March, April, May; Summer: June, July, August; Autumn: September, October, November; Day: 06:01 a.m. to 21:59 p.m.; Night: 10:00 p.m. to 06:00 a.m.; >6 lactations: 7th to 14th lactation; ITS: internal teat sealant, cfu: colony forming units

#### Herd- and cow-level factors affecting protein, fat and lactose concentrations

Tables 5 and 6 summarize the results of herd-level and cow-level factors affecting protein concentrations.

**Table 5:** Overview on the investigated herd-level factors influencing colostrum protein concentration. The 307 included colostrum samples originated from Simmental dairy cows. Protein concentration was measured at the Institute of Food Hygiene, Veterinary Faculty at Leipzig University applying standardized laboratory methods as described by the German Industry Standard (Deutsche Industry Norm, DIN). Additional information (herd-level factors) was gathered by an online questionnaire and an accompanying questionnaire

Herd-level category	Factor	N total samples	Mean protein	95% Confidence Interval		P value
				Lower CI	Upper CI	
Farm size	≤ 10 ^a^	15	11.32	9.08	13.57	< 0.01*
	11 to 20 ^b^	71	12.00	10.45	13.54	
	21 to 30 ^c, d^	69	13.53	12.24	14.83	
	31 to 40 ^a, b,c, e^	71	15.36	14.04	16.68	
	≥ 41 ^d, e^	81	11.71	10.38	13.04	
Udder cleaning before colostrum harvest	No	8	10.99	8.77	13.20	0.01*
	Yes	299	14.59	14.12	15.05	
District	Tennengau ^a, b,c^	19	15.04	13.35	16.73	< 0.01*
	Lungau ^a, d^	24	11.43	9.77	13.09	
	Pinzgau ^d, e,g^	81	13.97	12.77	15.18	
	Flachgau/SalzburgStadt^b, e,f^	132	12.46	11.21	13.72	
	Pongau ^c, f,g^	51	11.03	9.77	12.29	
Production regime	Conventional	107	13.04	11.71	14.37	0.15
	Organic	200	12.53	11.41	13.66	
Operation type	Full-time farmer	200	11.97	10.57	13.38	0.03*
	Part-time farmer	107	13.60	12.27	14.93	

Significant P values (*P* < 0.05) are highlighted with an asterisk *. Herd-level factors which were significantly different within the category are highlighted with superscript letters

**Table 6:** Overview on the differences in colostrum protein concentration at the cow-level. The 307 included colostrum samples originated from Simmental dairy cows. Protein concentration was measured at the Institute of Food Hygiene, Veterinary Faculty at Leipzig University applying standardized laboratory methods as described by the German Industry Standard (Deutsche Industry Norm, DIN). Additional information (herd-level factors) was gathered by an online questionnaire and an accompanying questionnaire. Additional information (herd-level factors) was gathered by an online questionnaire and an accompanying questionnaire

Cow-level category	Factor	N total samples	Mean protein	95% Confidence Interval		P value
				Lower	Upper	
Season of calving	Winter	103	10.87	8.66	13.08	< 0.01*
	Spring ^a^	47	10.20	8.09	12.30	
	Summer	63	11.56	9.02	14.11	
	Autumn ^a^	94	12.21	9.77	14.64	
Time of calving	Night	95	11.18	8.98	13.38	0.87
	Day	208	11.24	8.96	13.52	
Lactation number	2nd lactation ^a, b^	68	10.10	7.84	12.35	< 0.01*
	3rd lactation ^c, f^	51	9.69	7.16	12.23	
	4th lactation ^a, c,d^	32	12.11	9.81	14.40	
	5th lactation ^d, e^	27	9.92	7.47	12.37	
	6th lactation ^b, e,f^	14	13.83	10.97	16.69	
	> 6 lactations	26	11.61	9.28	13.94	
Dry period length	< 8 weeks	80	12.20	10.21	14.19	0.19
	8 to 12 weeks	126	12.39	10.48	14.29	
	> 12 weeks	10	9.04	4.90	13.19	
Dry off procedure	Antibiotic treatment ^a, b^	93	11.99	9.72	14.25	0.04*
	ITS ^A^	51	10.69	8.34	13.03	
	No medication ^b^	64	10.95	8.80	13.11	
Disease during the dry period	Yes	11	11.46	8.60	14.32	0.64
	No	289	10.96	9.00	12.92	
Colostrum leakage	Yes	45	9.22	6.55	11.89	< 0.01
	No	259	13.20	11.14	15.25	
Vaccination dam	Yes	49	11.76	9.36	14.17	0.05
	No	253	10.65	8.53	12.78	
Time to colostrum harvest	≤ 120 min ^a^	220	12.78	10.64	14.92	0.01*
	121 to 360 min ^a^	67	11.32	8.87	13.77	
	> 360 min	16	9.53	6.35	12.70	
Quantity colostrum harvest	0 to 3 L	145	11.44	9.11	13.77	0.05
	4 to 6 L	121	11.86	9.62	14.11	
	> 6 L	31	10.32	8.00	12.64	
Total plate counts	< 100,000 cfu/mL	96	11.75	9.42	14.08	n. a.
	not assessable	33	10.31	7.99	12.63	
	≥ 100,000 cfu/mL	43	11.57	9.37	13.77	
Coliform counts	< 10,000 cfu/mL ^a^	154	11.75	9.87	13.62	0.04
	not assessable ^a^	11	10.20	7.91	12.48	
	≥ 10,000 cfu/mL	7	11.69	8.10	15.28	

Significant P values (*P* < 0.05) are highlighted with an asterisk *. Herd-level factors which were significantly different within the category are highlighted with superscript letters. Winter: December, January, February; Spring = March, April, May; Summer = June, July, August; Autumn = September, October, November; Day = 06:01 a.m. to 21:59 p.m.; Night = 10:00 p.m. to 06:00 a.m.; >6 lactations = 7th to 14th lactation; ITS = internal teat sealant, cfu = colony forming units

Detailed calculation results of herd- and cow-level factors on fat and lactose concentrations are summarized in the Additional File 2. On a herd-level, the factor district had a significant impact on colostrum fat concentrations. Colostrum samples from the district of Lungau showed significantly (*P* = 0.03) higher mean fat concentrations (7.8%) in comparison to colostrum samples from Flachgau/Salzburg Stadt (4.91%). On a cow-level, the factors number of lactations, dry-off procedure and time to colostrum harvest had a significant impact on colostrum fat concentrations. Cows in their second lactation revealed higher (5.28%) fat concentrations than cows in their fifth lactation (3.74%). Cows receiving no dry-off medication showed higher fat concentrations (5.23%) in comparison to cows receiving an ITS (4.06%). Cows milked within 120 min after calving had higher fat concentrations (5.34%) versus cows milked between 121 and 360 min after calving (4.33%). On a herd-level the colostrum lactose concentrations were significantly different between the districts and samples originating from the district of Pongau showed significantly higher values (2.67%) in comparison to colostrum samples from Lungau (2.38%) or Pinzgau (2.28%).

## Discussion

This study is the first large scale attempt to investigate bovine colostrum samples originating from dairy cows from small scale farms in the federal state of Salzburg, Austria. The present study was carried out to describe factors that are associated with Brix, protein, fat and lactose concentration in dairy cow colostrum from Salzburg. Therefore, 1,050 colostrum samples from 72 dairy farms were analyzed. Further, 307 colostrum samples from pure-breed Simmental cows from 39 dairy farms were further analyzed to assess protein, fat and lactose concentrations. Another study carried out by our research group in the federal district of Salzburg, Austria, including 250 calves showed that 37.2% were categorised with FTPI whereby the herd-level prevalence varied severely between farms. The number of calves receiving a sufficient colostrum supply was 84.6% for the best farm and 26.7% for the worst farm [3]. This stresses the importance of further investigations and improvements in this area.

### Questionnaire results

In the field study convenience sampling was carried out but the gained results give a good overview on the farm structure in the federal state of Salzburg. In the present study, 46 (63.9%) organic and 26 (36.1%) conventional farms participated, respectively. Salzburg has 3,270 dairy farms, with 2,017 (61.7%) organic producers [26]. The number of organic farms was only slightly overrepresented, which was also the case in an online survey on colostrum management in Austria carried out by our group recently [26]. Additionally, the number of part-time farmers was high, with 31 (44.4%) which is also typical for the small-scale farming structure in Salzburg [29]. On average 34 cattle (year 2021) were kept on Austrian cattle farms [30]. The number of dairy cows per farm is small with 25.2% of the farms housing ≤ 10 dairy cows, 36.4% keeping between 11 and 20 dairy cows, 15.6% between 21 and 30 cows and 22.8% more than 31 dairy cows [26]. The questionnaire on colostrum management procedures revealed that only 7 (9.7%) of the 72 farmers use a device to test colostrum quality such as a refractometer or a hydrometer to test colostrum quality before delivering it to the calf. In a survey conducted in Austria in 2015 (1,501 responses) similar results were published whereof only 19.0% of the small farmers (≤ 20 cows) stated to check colostrum quality before delivering it to the calf. Of these 19%, only 2.5% used a colostrometer and 97.5% performed visual inspection, respectively [31]. This indicates that the number of small-scale farmers testing colostrum quality increased in the last years, but there is still need for considerable improvement. Within the present project the farmers had the opportunity to acquire a Brix refractometer, therefore the number of farmers testing colostrum quality increased. In total, 84.7% of the farmers feed single-dam colostrum to the calf independently of the IgG concentration and no farmer is pooling colostrum from multiple cows. A study conducted in Ireland showed that feeding single-dam colostrum improves the calf’s immunity through increased serum IgG levels. Nevertheless, the most important factor is that the amount of IgGs provided to the calf is high and subsequently the apparent efficiency of absorption [32]. In the present online questionnaire, 54.2% (39 farms) of the 72 farmers answered that they harvest colostrum within one hour after parturition. The assessment of the accompanying questionnaire of the collected colostrum samples showed that 52.0% (529 samples) of the colostrum samples were collected within one hour after parturition. In the present online questionnaire, 67 (93.1%) of the 72 farmers noted that they theoretically harvest the first colostrum within six hours. According to the accompanying questionnaire, 92.0% of the colostrum samples (936 samples) were collected within six hours. In the online questionnaire 39 (54.2%) of the farmers stated that they feed the calf within one hour after birth and 91.7% within six hours after birth. At the calf-level, 455 calves (45.6%) were fed within one hour and 939 (94.2%) within six hours after birth. It is well known that people tend to give the correct answer (knowing what they should do) instead of giving the true answer in questionnaires. The present results indicate that theory (online questionnaire results) and practice (colostrum sample collection results) correspond well.

### Colostrum Brix, fat, protein and lactose concentrations

Colostrum constituents, especially IgG concentration, varies significantly between herds which corroborates the substantial effect of herd-specific management factors [9]. In the present study, the number of samples with good and poor-quality colostrum in terms of Brix% was 517 (49.5%) and 528 (50.5%), respectively. This is comparable to the results from other reports for instance from Ireland (1,239 samples; good quality in 56% using a cut-off of 50 mg/mL IgG; poor quality in 44.0%, using an IgG ELISA as detection method) and the USA (827 samples; good quality in 70.6% using a cut-off of 50 mg/ml IgG, 29.4% poor quality, using RID as detection method) [9, 23]. An interesting finding was that conventional farms had significantly better colostrum qualities in terms of Brix% than organic producing farms (22.51% versus 21.06%). No difference was found in the mean protein concentration between conventional and organic farms (13.04% versus 12.53%). The number of studies comparing colostrum quality originating from organic and conventional farms are limited. This finding needs to be further investigated since potential explanations, such as organic farmers are primarily small-scale (< 20 cows/farm) and part-time, do not explain the findings since colostrum samples from part-time farmers showed a higher Brix% in comparison to full-time farms (mean 22.26% versus 21.31%). Since no sample size calculation was carried out the results should not be overinterpreted. For instance, some organic farms collected > 30 colostrum samples and this may have biased the outcome. Additionally, the feeding intensities (amount of roughage in the ration, amount of concentrate feed, micronutrients) differ between the production types.

The implemented methodology of IgG assessment varies between studies. In the present investigation we implemented the digital Brix refractometer and a cut-off of > 22% Brix which has a high diagnostic accuracy in terms of sensitivity and specificity, with high correlation between IgG and Brix scores [33]. Due to time and resource issues the gold standard (RID) was not carried out. The protein concentration determined by the Kjeldahl method and the Brix% determined by the digital Brix refractometer showed a high correlation in the present study of *r* = 0.91. This is in accordance with a study reporting a correlation of *r* = 0.83 for Brix% with IgG and *r* = 0.98 for Brix% with protein concentration [34]. The Brix% and the protein concentration increased with increasing parity which was evident in the cow-level analysis. Additionally, in the present study autumn and winter calving dairy cows had higher Brix% in comparison to cows calving in summer or spring. The samples originating from the district of Lungau showed a mean Brix% of 23.33%. It needs to be mentioned that all dairy farms in this district are located above 1000 m (3000 ft) sea level and due to the farming structure (alpine transhumance) the cows are primarily seasonal calving herds. Therefore, the climatic conditions for the dairy cows in this district are more favourable than for cows in e.g., Flachgau/ Salzburg Stadt. A study conducted in Northern Greece showed the same results that the cows had the highest colostrum protein concentration in autumn and winter [35]. The same findings were described [9] in Northern Ireland where cows showed the highest IgG concentrations in winter. It is well known that the Temperature-Humidity-Index and further heat stress plays an important role in dairy production systems. Nevertheless, further investigations are necessary to determine the cause-effect relationship since the findings in field studies on the effect of heat stress on colostrum quality and quantity are contradictory [10, 12]. In the present study, the time interval between calving and colostrum collection had a significant impact on colostrum quality in terms of Brix%, fat and protein concentration whereof the earlier the collection the higher the concentrations. These findings are in accordance with a study from Ireland including 21 commercial dairy farms and from Greece including 10 dairy farms [9, 35]. This has also been described by others [36] where models were created to predict colostrum IgG including multiparous Jersey cows. Additionally, the previous lactation milkfat % contributed the most toward increasing IgG concentration [36] Cows producing less colostrum had higher protein concentrations, this has been described by multiple authors whereby it is not feasible to give a reliable cut-off (litres of colostrum/colostrum quantity produced) for good or poor colostrum quality [10, 35, 37]. Colostrum quality was better in cows showing a colostrum quantity of ≤ 8.75 L using a classification and regression tree analysis with colostrum quality as the outcome variable. If colostrum quality was below this cut-off, parity had an influence on colostrum quality [10]. A study conducted in Northern Greece showed that colostrum quality was significantly higher in cows having ≤ 4 kg of colostrum yield. Overall, the fat, protein and lactose concentrations were similar to another investigation where the lower and upper fat, protein and lactose quartiles were 4.1% and 8.3%, 11.6% and 16.6% and 2.3 and 3.1%, respectively [9]. Previous studies focusing on the factors affecting colostrum fat, protein and lactose concentrations including more than 1,000 Holstein cows found that cows in their first lactation had significantly higher fat concentrations in comparison to cows with greater parities [9, 35]. In the present study the results were similar, the higher the number lactations the lower the colostrum fat concentration. Further it needs to be pointed out, that the colostrum samples from the present study originated predominantly from Simmental cows and the majority of other studies were carried out with samples from Holstein cows. Beside IgGs, proteins, fat and lactose colostrum contains multiple other essential constituents such as cytokines, growth factors, mRNA, oligosaccharides, maternal leucocytes, vitamins, minerals, hormones and non-specific antimicrobial factors [20]. What role some of the colostrum constituents play on calf health is still not completely understood, nor how processing techniques such as freezing and heat-treatment, impact these colostrum components. For instance, postharvest heat-treatment and freezing of colostrum eliminated viable colostral leukocytes and affected microRNA abundance and complement activity [38]. Therefore, further research is necessary in this area.

### Limitations

The field study was designed to investigate colostrum Brix% and not to investigate fat and lactose concentrations, therefore some herd- and cow-level factors could not be determined for these constituents. The assessment of a subset of colostrum samples for the analysis of the milk constituents was included as a pilot project to investigate the range of fat, protein and lactose concentrations of pure-breed dual-purpose Simmental breed. In future, a sample size calculation needs to be carried out beforehand to increase the external validity of the results. Some herd-level factors were found to have a significant impact on Brix%, protein, fat or lactose concentrations. For instance, the federal district where the farm was located. Since the farming structure varies widely within the federal state of Salzburg these results are presumably indirect effects which need to be further clarified. The results from a previous investigation by our group showed that part-time farms are smaller (< 20 dairy cows/farm), the farms have a lower milk yield per cow per year (< 7,500 L) and the herd-management practices are significantly different [26]. This might have impacted the present results and therefore the findings at the herd-level should not be overinterpreted. The farmers were trained in the sampling method following the SOP provided by the authors. In field studies including farmers there is always a chance that the SOP is not followed carefully. This point needs to be mentioned as limitation. Additionally, some cow-level factors were not further specified (no standard operating procedure) for the farmer, such as “hand milking” or “*ante partum* colostrum leakage”. Therefore, it cannot be excluded that there were variations within the groups. Some cows calved unassisted during the night in the maternity pen and the calves were separated as soon as the farmer noticed. Therefore, it cannot be excluded that the calves already suckled colostrum from the dam, influencing the measured Brix values from the collected colostrum. Also, the fact that 34% of the cows calved during the night might have biased the results since the time lag to colostrum harvest might be longer during nighttime. The number of samples per farm differed significantly (1 to 54 samples) since participation was on a voluntary basis (convenience sample) and no power calculation or sample size calculation was carried out before the study. Therefore, it needs to be stressed that the external validity is limited.

## Conclusions

In summary, field investigations such as the presented study raises the awareness for calf management especially colostrum management. The goal must be to achieve > 40% of calves with excellent passive transfer which can be achieved by feeding high quality, in terms of IgG/L and bacterial contamination, and high quantity colostrum as quickly as possible after birth.

The results confirmed that currently recognized cow-level factors such as season of calving, parity, colostrum leakage and time lag between calving and colostrum collection has a significant impact on colostrum quality in terms of Brix% and protein concentration, respectively. It needs to be stressed that many calves will apparently receive poor quality colostrum. Action is needed to transfer current knowledge into practice and to motivate farmers to implement a good colostrum management.

## Electronic supplementary material

Below is the link to the electronic supplementary material.

Supplementary Material 1

Supplementary Material 2

## Data Availability

Data are available within the article or in its Additional Files.
