# Supplementary material for: Evaluation of factors associated with immunoglobulin, protein, fat and lactose concentrations in colostrum of dairy cows from Austria
Source: Acta Vet Scand. 2024 Dec 25;66:63. doi: 10.1186/s13028-024-00788-0 (PMC11670480; doi:10.1186/s13028-024-00788-0)
Supplement: Supplementary file 1 — Supplementary Material 1 [file 13028_2024_788_MOESM1_ESM.docx]

***Colostrum sample collection***

Colostrum samples were collected by the 72 farmers between November 2020 and January 2022. The farmers received at least ten colostrum sampling kits. One colostrum sampling kit included disposable gloves, a 50 mL sterile tube (Greiner Bio-One International GmbH, Kremsmünster, Austria) and a 15 mL sterile tube (Greiner Bio-One International GmbH, Kremsmünster, Austria). Additionally, a detailed colostrum sampling protocol (standard operating procedure - SOP) was added to each test kit for the farmers. The SOP is provided as Figure 1 (in German). Following calving, the colostrum was routinely harvested (milking machine or hand milking) by the farmers. Subsequently, the colostrum was sampled at the point of calf feeding. For example, for farmers feeding the colostrum using a feeding bucket, the sample was obtained directly from the feeding bucket. Two tubes (15 mL and 50 mL) were filled with colostrum wearing disposable gloves. Additionally, the farmer completed a detailed accompanying questionnaire for every single colostrum sample which included information regarding the dairy cow (number of lactations, dry period length, dry-off procedure, health issues during the dry period), parturition and colostrum harvest. The accompanying questionnaire is provided in Figure 2 (in German) and Figure 3 (in English). Colostrum leakage *ante partum* was defined as colostrum leaking during parturition and/or before first colostrum harvest. Additionally, detailed information on calving season (calving month), time of calving (night: 10:00 p.m. to 06:00 a.m.; day: 06:01 a.m. to 09:59 p.m.), time of colostrum harvest and time of colostrum delivery to the calf was gathered.

***Colostrum sample storage and logistics***

All samples were frozen on the farm in a freezer (minus 18 to 20 °C) until one of the authors picked up the colostrum samples. Subsequently, the frozen samples were transferred on ice in a polystyrene box via special medicinal logistics (MedLog) or by one of the authors (KL, NH, AP) to the diagnostic laboratory of the Clinical Center for Ruminant and Camelid Medicine, Vetmeduni Vienna. The samples were thawed in the refrigerator at 4 to 8 °C and prepared for further analysis.

**
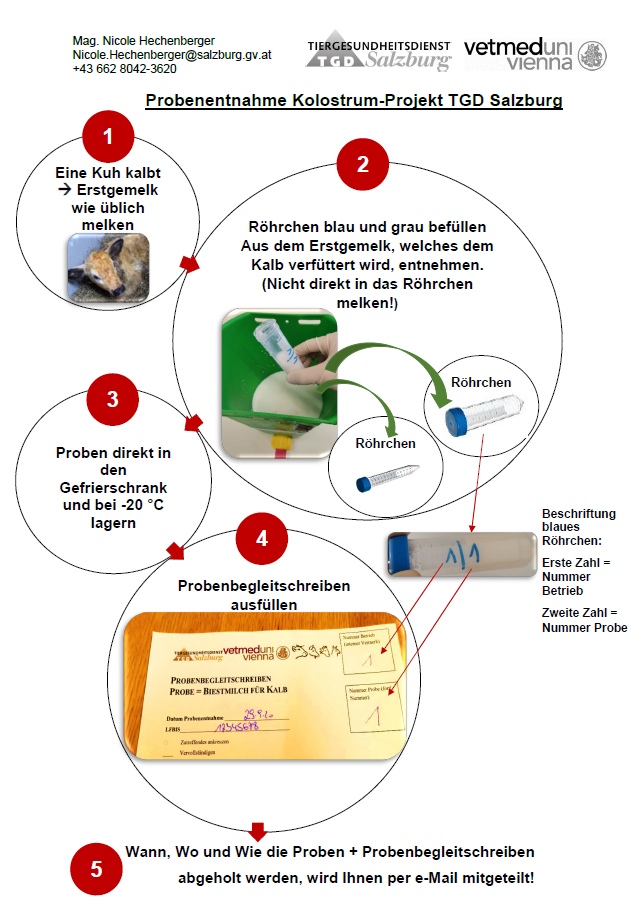
**

**Additional Figure 1:** The figure shows the standard operating procedure (SOP) for colostrum sample collection. The colostrum samples were collected by the farmers following this SOP. In advance they received a colostrum collection kit including disposable gloves (size large), a 50 mL tube (Greiner Bio-One International GmbH, Kremsmünster, Austria) and a 15 mL sterile tube (Greiner Bio-One International GmbH, Kremsmünster, Austria).

**
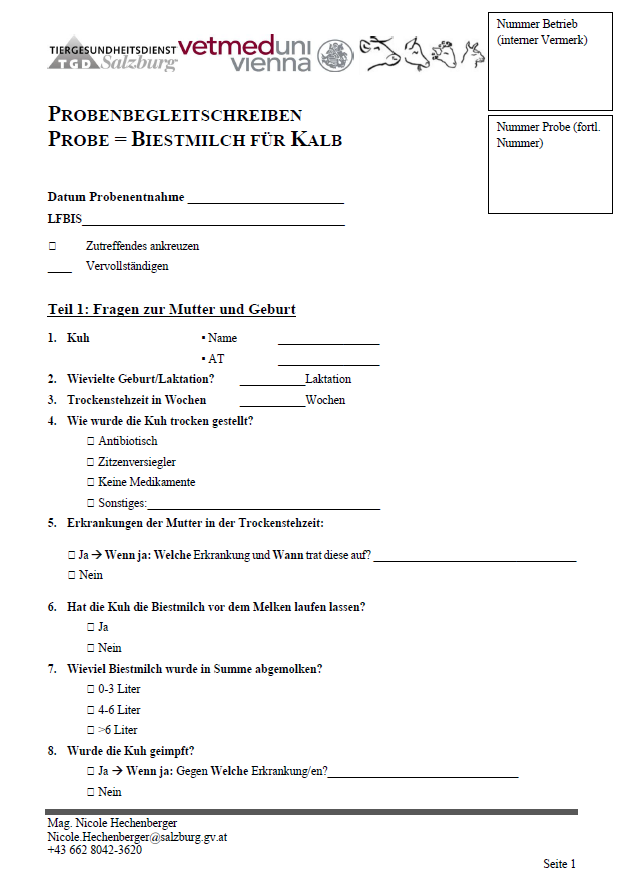
**

**Additional Figure 2:** Accompanying questionnaire gathering information on a cow- and calf-level.

**
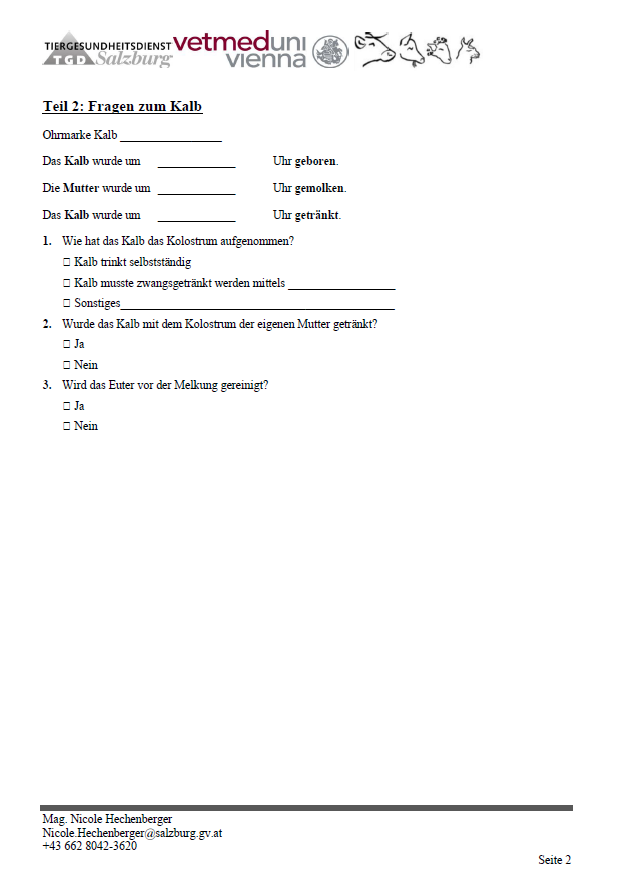
**

**Additional Figure 2 continued:** Accompanying questionnaire gathering information on a cow- and calf-level.

***
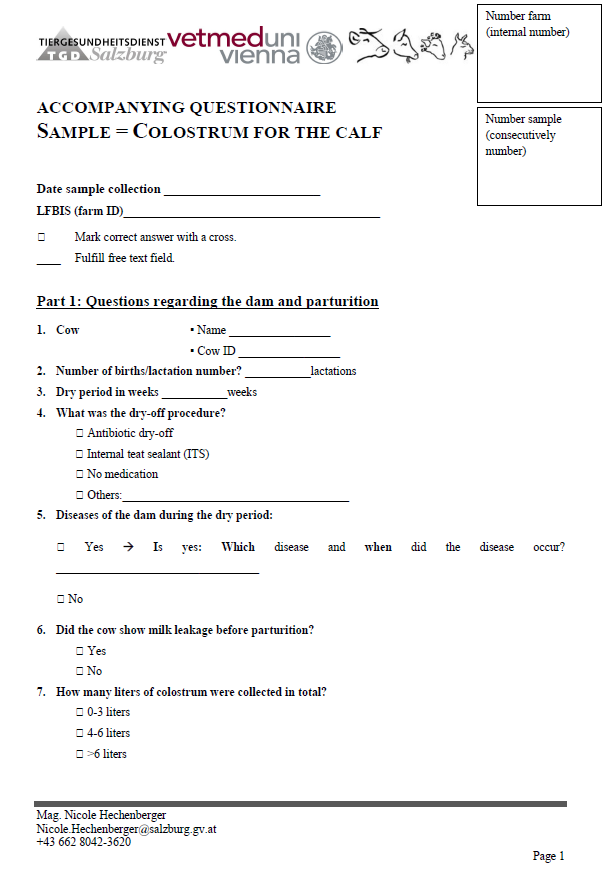
***

**Additional Figure 3:** The accompanying questionnaire was completed by the farmer for each colostrum sample.

***
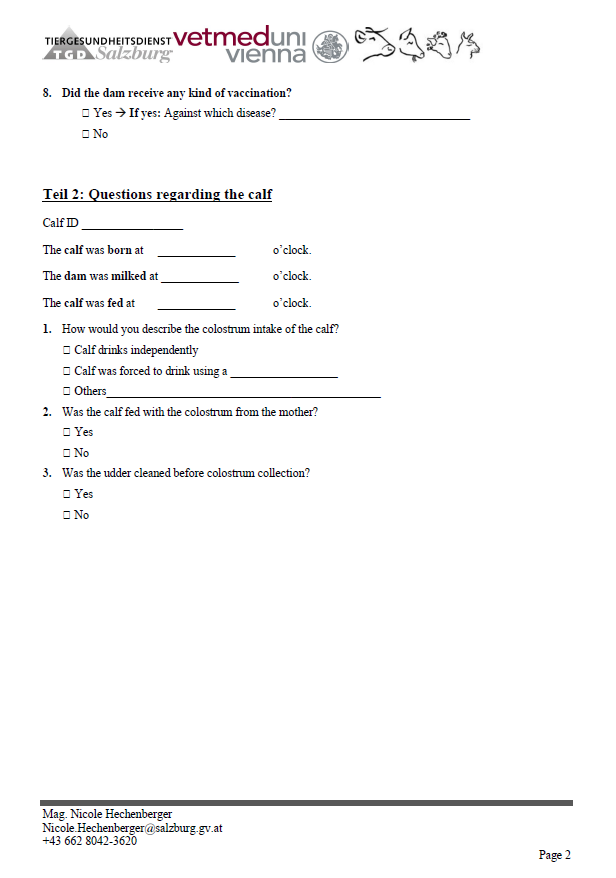
***

**Additional Figure 3 continued:** The accompanying questionnaire was completed by the farmer for each colostrum sample.
