# Supplementary material for: Evaluation of factors associated with immunoglobulin, protein, fat and lactose concentrations in colostrum of dairy cows from Austria
Source: Acta Vet Scand. 2024 Dec 25;66:63. doi: 10.1186/s13028-024-00788-0 (PMC11670480; doi:10.1186/s13028-024-00788-0)
Supplement: Supplementary file 2 — Supplementary Material 2 [file 13028_2024_788_MOESM2_ESM.docx]

**Additional Table 1:** Overview of fat, protein and lactose concentrations of 307 colostrum samples from Simmental cows.

| **Lactation number** | **Samples**  **N** | **Constituent**  **%** | **Minimum**  **%** | **Maximum**  **%** | **Percentile%** | | |
| --- | --- | --- | --- | --- | --- | --- | --- |
|  |  |  |  |  | **25** | **50** | **75** |
| **1** | 89 | % fat | 1.7 | 18.5 | 5.7 | 7.9 | 9.9 |
|  |  | % protein | 4.2 | 27.5 | 12.2 | 15.5 | 17.0 |
|  |  | % lactose | 0.7 | 4.3 | 2.0 | 2.2 | 2.6 |
| **2** | 68 | % fat | 1.2 | 14.4 | 3.7 | 5.3 | 7.3 |
|  |  | % protein | 5.6 | 20.2 | 12.1 | 13.1 | 15.5 |
|  |  | % lactose | 0.2 | 3.5 | 2.1 | 2.3 | 2.6 |
| **3** | 51 | % fat | 0.6 | 16.5 | 2.9 | 4.1 | 5.2 |
|  |  | % protein | 5.7 | 22.3 | 11.1 | 13.2 | 16.0 |
|  |  | % lactose | 1.4 | 5.0 | 2.1 | 2.4 | 2.8 |
| **4** | 32 | % fat | 0.5 | 9.2 | 3.2 | 4.2 | 5.3 |
|  |  | % protein | 10.1 | 21.9 | 13.7 | 15.1 | 16.2 |
|  |  | % lactose | 1.1 | 3.4 | 2.0 | 2.2 | 2.6 |
| **5** | 27 | % fat | 0.8 | 7.8 | 2.0 | 3.0 | 5.3 |
|  |  | % protein | 9.7 | 20.0 | 12.5 | 15.6 | 17.0 |
|  |  | % lactose | 1.7 | 3.2 | 2.0 | 2.3 | 2.7 |
| **6** | 14 | % fat | 2.5 | 14.7 | 4.2 | 4.7 | 6.1 |
|  |  | % protein | 7.3 | 22.8 | 12.5 | 16.2 | 19.8 |
|  |  | % lactose | 1.0 | 2.8 | 1.6 | 2.0 | 2.5 |
| **> 6** | 26 | % fat | 1.0 | 8.4 | 2.2 | 3.2 | 4.3 |
|  |  | % protein | 11.6 | 25.5 | 14.9 | 17.0 | 20.0 |
|  |  | % lactose | 1.3 | 2.9 | 1.8 | 2.1 | 2.4 |

**Additional Table 2:** Overview on the differences in colostrum fat concentration at the herd-level.

| **Herd-level**  **category** | **Factor** | **Mean Fat** | **95% Confidence Interval** | | **P value** |
| --- | --- | --- | --- | --- | --- |
|  |  |  | **Lower CI** | **Upper CI** |  |
| **Farm size** | ≤ 10 |  |  |  | n. a. |
|  | 11 to 20 | 5.38 | 3.76 | 7.00 |  |
|  | 21 to 30 | 5.51 | 4.29 | 6.72 |  |
|  | 31 to 40 | 4.73 | 3.52 | 5.94 |  |
|  | ≥ 41 | 6.43 | -9.81 | 22.68 |  |
| **Udder cleaning before colostrum harvest** | No | 5.18 | 2.72 | 7.65 | n. a. |
|  | Yes | 6.22 |  |  |  |
| **District** | Tennengau | 4.54 | 2.77 | 6.32 | 0.03* |
|  | Lungau^a^ | 7.80 | 5.46 | 10.13 |  |
|  | Pinzgau | 5.88 | 4.56 | 7.20 |  |
|  | Flachgau/Salzburg Stadt^a^ | 4.91 | 2.95 | 6.87 |  |
|  | Pongau | 5.37 | 4.01 | 6.73 |  |
| **Production regime** | Conventional | 6.21 | 4.39 | 8.03 | 0.22 |
|  | Organic | 5.19 | 3.87 | 6.52 |  |
| **Operation type** | Full-time farmer | 5.58 | 3.71 | 7.46 | 0.56 |
|  | Part-time farmer | 5.81 | 4.52 | 7.11 |  |

Significant P values (P<0.05) are highlighted with an asterisk *. Herd-level factors which were significantly different within the category are highlighted with superscript letters. The categories ‘farm size’ and ‘udder cleaning method’ were not assessable (n. a.) since the factor ‘level combinations’ (number of farms within the factor) was too low.

**Additional Table 3:** Overview on the differences in colostrum fat concentrations at the cow-level.

| **Cow-level category** | **Factor** | **Mean Fat** | **95% Confidence Interval** | | **P value** |
| --- | --- | --- | --- | --- | --- |
|  |  |  | **Lower** | **Upper** |  |
| **Season of calving** | Winter | 4.41 | 2.60 | 6.21 | 0.50 |
|  | Spring | 4.15 | 2.29 | 6.00 |  |
|  | Summer | 4.63 | 2.42 | 6.83 |  |
|  | Autumn | 4.96 | 2.88 | 7.05 |  |
| **Time of calving** | Night | 4.61 | 2.76 | 6.45 | 0.73 |
|  | Day | 4.47 | 2.58 | 6.36 |  |
| **Lactation number** | 2^nd^ lactation ^a^ | 5.28 | 3.33 | 7.23 | 0.01* |
|  | 3^rd^ lactation | 3.98 | 1.95 | 6.01 |  |
|  | 4^th^ lactation | 5.42 | 3.73 | 7.11 |  |
|  | 5^th^ lactation ^a^ | 3.74 | 1.84 | 5.65 |  |
|  | 6^th^ lactation | 4.87 | 2.09 | 7.64 |  |
|  | > 6 lactations | 3.93 | 2.09 | 5.77 |  |
| **Dry period length** | < 8 weeks | 3.97 | 2.12 | 5.81 | 0.54 |
|  | 8 to 12 weeks | 4.39 | 2.76 | 6.02 |  |
|  | > 12 weeks | 5.25 | 1.76 | 8.75 |  |
| **Dry off procedure** | Antibiotic treatment | 4.32 | 2.40 | 6.25 | 0.03* |
|  | ITS ^a^ | 4.06 | 2.18 | 5.94 |  |
|  | No medication ^a^ | 5.23 | 3.37 | 7.08 |  |
| **Disease during the dry period** | Yes | 4.08 | 1.56 | 6.59 | 0.38 |
|  | No | 5.00 | 3.42 | 6.58 |  |
| **Colostrum leakage** | Yes | 5.03 | 2.89 | 7.16 | 0.12 |
|  | No | 4.05 | 2.37 | 5.72 |  |
| **Vaccination dam** | Yes | 4.65 | 2.63 | 6.67 | 0.64 |
|  | No | 4.42 | 2.69 | 6.16 |  |
| **Time to colostrum harvest** | ≤ 120 minutes ^a^ | 5.34^a^ | 3.55 | 7.13 | 0.02* |
|  | 121 to 360 minutes ^a^ | 4.33^a^ | 2.34 | 6.32 |  |
|  | > 360 minutes | 3.94 | 1.12 | 6.75 |  |
| **Quantity colostrum harvest** | 0 to 3 litres | 4.07 | 2.16 | 5.99 | 0.06 |
|  | 4 to 6 litres | 5.16 | 3.35 | 6.97 |  |
|  | >6 litres | 4.38 | 2.41 | 6.35 |  |
| **Total plate counts** | <100,000 cfu/mL | 4.80 | 2.93 | 6.67 | 0.15 |
|  | not assessable | 4.86 | 2.93 | 6.78 |  |
|  | ≥100,000 cfu/mL | 3.95 | 2.06 | 5.84 |  |
| **Coliform counts** | <10,000 cfu/mL | 4.27 | 2.61 | 5.93 | 0.77 |
|  | not assessable | 4.63 | 2.41 | 6.84 |  |
|  | ≥10,000 cfu/mL | 4.71 | 2.53 | 6.89 |  |

Significant P values (P<0.05) are highlighted with an asterisk *. Herd-level factors which were significantly different within the category are highlighted with superscript letters.

**Additional Table 4:** Overview on the differences in colostrum lactose concentration at the herd-level.

| **Herd-level**  **category** | **Factor** | **Mean Lactose** | **95% Confidence Interval** | | **P value** |
| --- | --- | --- | --- | --- | --- |
|  |  |  | **Lower CI** | **Upper CI** |  |
| **Udder cleaning before colostrum harvest** | No | 2.54 | 2.22 | 2.85 | 0.13 |
|  | Yes | 2.29 | 2.24 | 2.35 |  |
| **District** | Tennengau | 2.38 | 2.10 | 2.66 | < 0.01 |
|  | Lungau ^a,b^ | 2.38 | 2.21 | 2.55 |  |
|  | Pinzgau ^c^ | 2.28 | 2.12 | 2.45 |  |
|  | Flachgau/SalzburgStadt ^a,d^ | 2.37 | 2.20 | 2.54 |  |
|  | Pongau ^b,c,d^ | 2.67 | 2.50 | 2.84 |  |
| **Production regime** | Conventional | 2.34 | 2.17 | 2.52 | 0.02 |
|  | Organic | 2.49 | 2.32 | 2.65 |  |
| **Operation type** | Full-time farmer | 2.42 | 2.25 | 2.59 | 0.89 |
|  | Part-time farmer | 2.41 | 2.23 | 2.59 |  |

Significant P values (P<0.05) are highlighted with an asterisk *. Herd-level factors which were significantly different within the category are highlighted with superscript letters. The category farm size was not assessable and excluded from the table.
